# Supplementary material for: Crawling and Gliding: A Computational Model for Shape-Driven Cell Migration
Source: PLoS Comput Biol. 2015 Oct 21;11(10):e1004280. doi: 10.1371/journal.pcbi.1004280 (PMC4619082; doi:10.1371/journal.pcbi.1004280)
Supplement: S1 Code — (ZIP) [file pcbi.1004280.s012.zip › release/tst/doc/html/hull_8cpp.html]

Tissue Simulation Toolkit: hull.cpp File Reference


Tissue Simulation Toolkit
 0.1.4.1


- Main Page
- Namespaces
- Classes
- Files

- File List
- File Members

Functions

hull.cpp File Reference

`#include "hull.h"`

Include dependency graph for hull.cpp:

|  |  |
| --- | --- |
| Functions | |
| float | isLeft (Point P0, Point P1, Point P2) |
|  | |
| int | chainHull\_2D (Point \*P, int n, Point \*H) |
|  | |

## Function Documentation

|  |  |  |  |
| --- | --- | --- | --- |
| int chainHull\_2D | ( | Point \* | *P*, |
|  |  | int | *n*, |
|  |  | Point \* | *H* |
|  | ) |  |  |

References isLeft(), and Point::x.

Referenced by CellularPotts::Compactness(), and CellularPotts::DrawConvexHull().

|  |  |  |  |
| --- | --- | --- | --- |
| float isLeft | ( | Point | *P0*, |
|  |  | Point | *P1*, |
|  |  | Point | *P2* |
|  | ) |  |  |

inline

References Point::x, and Point::y.

Referenced by chainHull\_2D().


---

Generated on Thu Aug 14 2014 22:04:01 for Tissue Simulation Toolkit by  

 1.8.6
